# Supplementary material for: Evaluation of the Hepatotoxicity of the Zhi-Zi-Hou-Po Decoction by Combining UPLC-Q-Exactive-MS-Based Metabolomics and HPLC-MS/MS-Based Geniposide Tissue Distribution
Source: Molecules. 2019 Jan 31;24(3):511. doi: 10.3390/molecules24030511 (PMC6384998; doi:10.3390/molecules24030511)
Supplement: Supplementary file 1 [file molecules-24-00511-s001.pdf]

Table 1. Precision, accuracy, recovery and matrix effect of geniposide the assay method for in biological samples.

Biological samples	Nominal concentration (ng/mL)	Intra-day (n=6)		Inter-day (n=18)		Recovery (n=6) (%)	Matrix effect (n=6) (%)
		RSD (%)	RE (%)	RSD (%)	RE (%)		
Plasma	5.00	9.3	3.4	8.2	-0.5	-	-
	12.5	5.8	10.9	7.9	6.9	81.8	97.6
	400	3.1	4.7	5.5	7.0	84.0	111
	4000	2.8	0.6	5.6	3.2	81.2	96.6
Liver	5.00	7.3	-8.2	12.1	3.3	-	-
	12.5	4.5	9.7	5.5	12.0	95.9	119
	400	2.4	1.9	5.4	8.8	85.2	94.5
	4000	3.1	5.2	4.6	9.2	84.1	93.5

Table S2. Stability of geniposide in rat plasma and tissue homogenate under various storage conditions.

Biological samples	Nominal concentration (ng/mL)	Room temperature for 15 h*/12 h**(n=3)	Three freeze-thaw cycles (n=3)	-80ºC for 90 days (n=3)	Autosampler (8ºC) for 53 h (n=6)
		RE (%)	RE (%)	RE (%)	RE (%)
Plasma	12.5	14.1	7.2	12.5	11.7
	400	-	-	-	4.9
	4000	8.7	-0.3	7.2	0.0
Liver	12.5	8.5	3.7	11.5	15.7
	400	-	-	-	15.1
	4000	2.6	-6.5	2.5	14.6

*: plasma; **: liver.
